# The origin and early evolution of feathers: implications, uncertainties and future prospects

**DOI:** 10.1098/rsbl.2024.0517

**Published:** 2025-02-19

**Authors:** Xing Xu, Paul M. Barrett

**Affiliations:** ^1^ Institute of Vertebrate Paleontology and Paleoanthropology, Chinese Academy of Sciences, Beijing 100044, China; ^2^ Centre for Vertebrate Evolutionary Biology, Yunnan University, Kunming, Yunnan 650504, China; ^3^ Fossil Reptiles, Amphibians and Birds Section, Natural History Museum, Cromwell Road, London SW7 5BD, UK

**Keywords:** feathers, integument, fossils, developmental biology, evolution

## Abstract

As a defining feature of the clade, feathers are key to understanding bird biology. Discoveries of spectacular dinosaur and pterosaur fossils preserving feathers and feather-like integumentary appendages demonstrate trends of increasing complexity in gross morphology and microstructure through avemetatarsalian evolution, and the acquisition of complex flight feathers before the origin of birds. Moreover, this material shows some early feathers differed from modern feathers morphologically, ultrastructurally, biochemically and developmentally, revealing integumentary evolutionary pathways absent in modern taxa. These advances have changed conventional understanding of dinosaurs and impacted conceptions of both birds and feathers. However, it remains unknown if ‘true’ feathers originated at the base of Avemetatarsalia or within Theropoda. The former scenario implies multiple feather losses, the evolutionary and developmental mechanisms of which require investigation; the latter suggests pterosaurs and ornithischians independently evolved filamentous integumentary appendages, which might have shared genetic regulatory networks with theropod feathers. Answering these questions requires additional data on avemetatarsalian integument, particularly for sauropodomorphs, early diverging theropods and dinosaur outgroups, and more information on those taxa with known integumentary features. An integrative approach combining morphological, developmental, biochemical and taphonomic data, including extinct and extant taxa, is essential for a clearer understanding of feather origin and evolution.

## 1. Introduction

In modern birds, feathers are complex, epidermally derived appendages that perform an exceptionally wide array of functions integral to avian biology [1–3]. They differ from other epidermal appendages in having a set of unique biochemical, developmental and morphological features, including feather-type corneous beta-proteins (i.e. feather type β-keratins or Ф-keratins), a distal–proximal growth mode, a follicle for development, and hierarchical levels of branching structures (rachis, barbs and barbules), and some other features [4,5]. Most obviously, feathers provide the contoured, controllable aerodynamic surfaces that support sustained, powered flight, but other vital functions include (among others) detection of tactile stimuli; inter- and intraspecific communication and signalling; thermal insulation and shielding; waterproofing; nest construction; brooding; protection from parasites; and camouflage [1–3].

**Figure 1.**
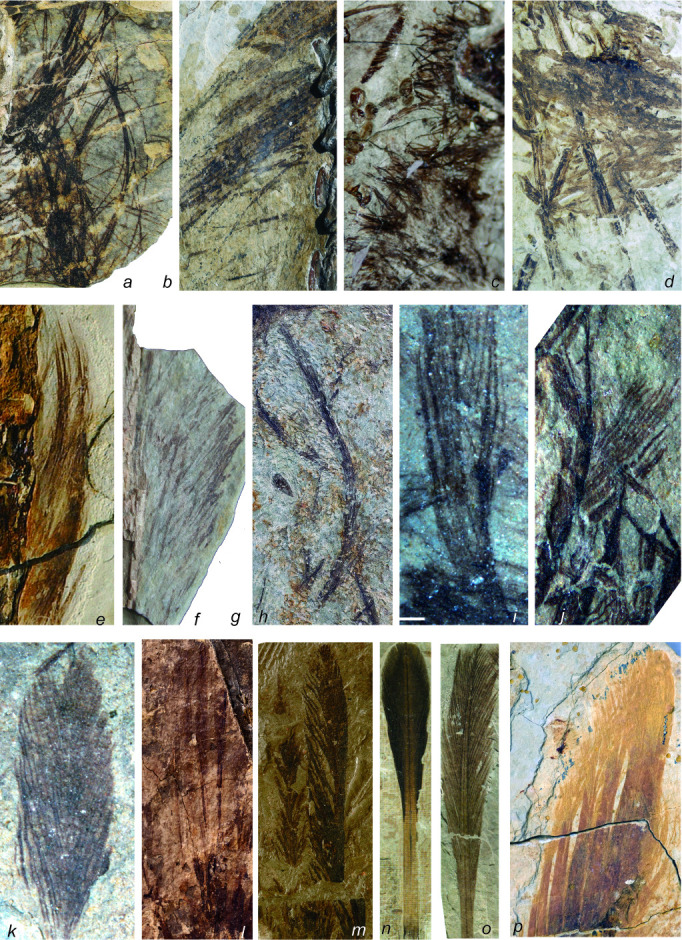
Feathers and feather-like integumentary structures among dinosaurs and pterosaurs. (*a*) Monofilamentous feather-like structures in the ornithischian dinosaur *Tianyulong*. (*b*) Monofilamentous feathers in the theropod dinosaur *Beipiaosaurus*. (*c*) Broad monofilamentous feathers in *Beipiaosaurus*. (*d*) Filamentous feather-like structures in a pterosaur specimen. Radially branched feathers in the theropod dinosaurs *Sinosauropteryx* (*e*) and *Anchiornis* (*f*). Bilaterally branched feathers in the theropod dinosaurs *Dilong* (*g*), *Sinornithosaurus* (*h*), and *Anchiornis* (*i*). (*j*) Pennaceous wing feathers with symmetrical vanes in *Anchiornis*. (*k*) Pennaceous tail feathers with symmetrical vanes in the theropod dinosaur *Similicaudipteryx*. (*l*) Pedal flight feathers with asymmetrical vanes in the theropod dinosaur *Microraptor*. Rachis-dominant tail feathers in the avialan *Confuciusornis* (*m*) and an enantiornithine bird (*n*). (*o*) Proximally ribbon-like tail feathers in *Similicaudipteryx*. Photographs not to scale.

**Figure 2.**
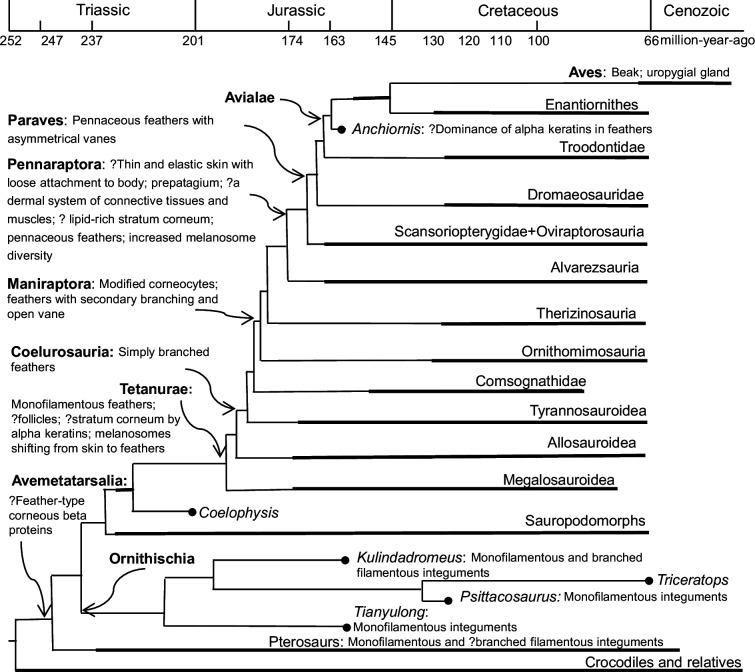
Distribution of known major integumentary features across a simplified avemetatarsalian phylogeny. ‘?’ indicates uncertainty regarding the presence of this particular feature in this group; features associated terminal taxa are likely to be widely distributed.

Until recently, feathers were regarded as uniquely avian—the *sine qua non* of what it was to be a bird [6]. This idea was so strongly held among naturalists that the mere presence of feather impressions around the skeleton of *Archaeopteryx* was enough to cement its status as the earliest-known bird, and the origins of birds, feathers and flight were considered closely correlated in most early narratives on bird evolution [6,7]. However, a series of spectacularly preserved fossil discoveries, primarily from the Early Cretaceous of China, revealed the presence of feathers (and other feather-like structures) in a variety of non-volant theropod dinosaurs (figure 1) [8–17], demonstrating conclusively that earlier models of bird evolution were wrong, as feathers clearly appeared prior to the origin of either birds or flight and must have had a deeper, dinosaurian ancestry (figure 2) [18–20]. These discoveries have changed our conventional understanding of what non-avian dinosaurs looked like, as well as dinosaurian biology, and they also significantly impacted the definitions of both birds and feathers [20,21]. For example, should we call all non-avialan pennaraptorans dinosaurs or birds [21]?

These discoveries, which occurred alongside advances in developmental biology that revealed the molecular and cellular mechanisms underlying feather formation (e.g. [4,22–26]), have also fuelled much new work on feather evolution, undermining previous hypotheses for feather origin and leading to controversy over exactly when, and why, these structures first appeared. Here, we provide a summary of the current consensus on feather origins, point out areas that remain contentious or poorly known and suggest potential avenues for resolution of these issues.

## 2. Feather origins and early evolution: the current consensus

The theropod fossil record shows clearly that the first feathers were simple and filamentous, while more complex feathers, such as those displaying branching structures, appeared later in evolutionary terms, with flight feathers, including those with asymmetrical vanes, evolving before the appearance of birds (figures 1 and 2) [18–20,23]. The phylogenetically earliest theropod feathers known currently were documented in the megalosauroid *Sciurumimus*, and these have a simple, monofilamentous morphology [15,27]. Monofilamentous feathers are also present in the tyrannosauroid *Yutyrannus* [28], the therizinosauroid *Beipiaosaurus* [29] and the possible compsognathid *Juravenator* [13,27], although in the former two taxa, the monofilaments are proportionally broad, probably representing specialized ornamental feathers [20]. The second stage of theropod feather evolution is documented by either radially or bilaterally branched feathers in various early diverging coelurosaurian theropods [30], such as *Dilong* [12] and *Sinosauropteryx* [31]. Pennaceous feathers with symmetrical vanes and asymmetrical vanes appeared at the bases of Pennaraptora and Paraves, respectively, representing the third and fourth evolutionary stages for theropod feathers (e.g. [20,32]).

At the microscopic level, different melanosome types have been reported in some theropod taxa, though geochemical evidence has yet to be presented to confirm the melanosome identifications of these spherical or rod-like structures. Phaeomelanosomes have been reported in simply branched feathers of *Sinosauropteryx* and *Beipiaosaurus* [33]; whereas both phaeomelanosomes and eumelanosomes are known in several early diverging pennaraptorans, such as *Yi, Anchiornis* and *Microraptor* [34–36]. These melanosomes display a trend of increasing diversity towards the origin of birds, and a significant increase occurs at the base of Pennaraptora [37].

At least some feather developmental data are informative for reconstructing feather origins and early evolution, and several developmental models have been proposed [4,23–26,38]. These models suggest an evolutionary sequence of: monofilamentous feathers; branched feathers with or without secondary branching (i.e. presence of barbules); branched feathers with a rachis; and pennaceous feathers. Feather tracts might have appeared at the earliest evolutionary stage for feathers given the earlier development of feather tracts with respect to feather buds and follicles [39], but their appearance has also been suggested to be associated with pennaceous feathers [40]. Although modern bird feathers are always associated with a follicle [4], it has been proposed that follicles might have had a later origin, perhaps after the appearance of filamentous morphology [40]. At the molecular level, various genes and their expression patterns are known to be responsible for differing morphologies in modern feather diversity and their distributions [41]: *Wnt-β-catenin, FGF* and *BMP* are involved in determining feather types and their distributions [42–44]; *BMP*, *noggin* and *SHH* participate in the formation of the rachis and barbs [39,45,46]; *Wnt3a* contributes to rachis positioning [43]; *Wnt2b* assists in vane formation [47]; and retinoic acid is involved in vane asymmetry formation [48]. These molecular and developmental mechanisms are generally consistent with the embryological and morphological sequences of barb to rachis and of radially symmetric feather to bilaterally symmetric feather.

The morphology and phylogenetic distribution of theropod feathers also imply a functional evolutionary sequence (see figure 2). Monofilamentous feathers in early diverging tetanuran theropods are most likely to have functioned in insulation, camouflage or display, with none of these functions being mutually exclusive. A more specialized communication/signalling function might have appeared early in theropod feather evolution given that the early diverging tetanuran *Sciurumimus* displays a ‘feather crest’ along the tail, and early diverging coelurosaurian theropods, such as *Yutyrannus* and *Beipiaosaurus*, have some specialized ornamental feathers [40,49]. Significantly, increased melanosome diversity at the base of Pennaraptora might be an indicator of an increased communication/signalling function [33–35,50], although it has also been suggested to indicate a physiological shift [37] or a shift to more diverse melanin-based colours and structural colours [51], none of which are mutually exclusive explanations. Furthermore, pterosaur melanosomes support a signalling function for early feathers [52], assuming that the monofilamentous integumentary appendages in this group are indeed feathers (see below). Flight function might have appeared around the cladogenesis of Pennaraptora [53,54], given that beside flight feathers, several other flight-related features evolved at the base of this group, including long, robust arms, cerebral expansion and visual region elaboration, as well as elevated metabolic rates [53].

## 3. Areas of conflict and potential resolution

While there is general agreement that the integumentary appendages present in tetanuran theropods are homologous (e.g. [18,40,55]), there is less consensus regarding those present in ornithischian dinosaurs and pterosaurs [52,55–58]. Monofilamentous integumentary appendages are known in only three ornithischians: the heterodontosaurid *Tianyulong* [59], the early diverging neornithischian *Kulindadromeus* [56,60] and the ceratopsian *Psittacosaurus* [61,62]. *Kulindadromeus* also possesses simply branched filamentous integumentary appendages and a range of other unusual epidermal structures including the one with multiple filaments arising from a basal plate [56,60]. Among non-dinosaurian archosaurs, which include crocodilians and a range of other Mesozoic taxa, only pterosaurs have monofilamentous integumentary appendages [63,64] and some even display simply branched integumentary appendages [52,57].

Monofilamentous integumentary appendages in ornithischians and pterosaurs have been considered primitive feathers by most authors, and together with theropod feathers, their discoveries have led to the hypothesis that feathers originated at the base of either Dinosauria or Avemetatarsalia [29,40,57,59]. However, some studies support a tetanuran theropod origin hypothesis and regard the filamentous integumentary appendages in ornithischians and pterosaurs as either specialized scales or appendages that are not homologous with theropod feathers but that share morphological similarities such as filamentous shape [55,58].

Current data indicate that the filamentous integumentary appendages in ornithischians and pterosaurs are more similar to primitive feathers than to specialized scales [20,52,57], including the modified epidermal scales seen in several extinct and extant reptiles and the midline scale frills in some dinosaurs. In particular, no reptilian scale displays the branched morphology present in theropods and some of the integumentary appendages seen in ornithischians and pterosaurs. Collectively, these features provide evidence for the identification of the integumentary appendages of ornithischians and pterosaurs as feathers, based on similarity criteria. However, using these same criteria for primary homology, some of the unusual integumentary structures present in the ornithischian *Kulindadromeus* (such as the one with multiple filaments originating from a basal plate) have no analogues among theropods or pterosaurs and their identifications as feathers are less secure.

Admittedly, the branched morphology reported in some ornithischians and pterosaurs requires additional documentation to establish that this feature is genuine given that fossilization can produce artificial morphologies, including branching patterns [65]. More importantly, it is known that all modern feathers grow from a follicle, which is considered a key criterion for identifying modern feathers [4]. For example, wild turkey ‘beards’ are epidermal outgrowths with both feather-type corneous beta proteins and branching morphology but without an associated follicle, and thus they are not considered true feathers. If the filamentous integumentary appendages in ornithischians and pterosaurs can be shown to have grown from a follicle, they are clearly feathers; if not, alternative definitions, recognizing that follicles might be derived structures, might still allow them to be considered as feathers (see §4).

In terms of secondary homology (i.e. synapomorphy), it is difficult to test whether feathers are plesiomorphic for Dinosauria or Avemetatarsalia due to the incompleteness of the fossil record, both with respect to taxon sampling and fossil preservation quality. For example, we currently lack information on the integument of early diverging theropods and sauropodomorphs and have no data on other key avemetarsalian groups, including lagerpetids, silesaurids and aphanosaurs, due to the scarcity of Middle Triassic to Early Jurassic terrestrial deposits with the potential for soft-tissue preservation. The early stratigraphic occurrences of the aforementioned taxa (as well as early occurring pterosaurs and ornithischians) would have major effects on probabilistic modelling of the timing of feather origins, as well as on the phylogenetic optimizations of integumentary features [52,55,57,58]. Currently, some analyses suggest that feathers originated as early as 245 Ma at the base of Avemetatarsalia [52,57], while others consider feathers to have originated much later, within Theropoda (with the filamentous structures in pterosaurs and ornithischians considered as independent acquisitions) [58] (see figure 2).

The incompleteness issue is highlighted by our restricted knowledge of the distribution of filamentous structures on the body of *Psittacosaurus*. This taxon is represented by hundreds of well-preserved specimens, but only one of these preserves monofilamentous integumentary appendages [61]. This suggests that some otherwise scaled dinosaurs might have had similar structures but only on certain parts of their bodies or, conversely, that they arose in some taxa in isolation under rare circumstances. Furthermore, ornithischians and pterosaurs with known filamentous integumentary appendages are all small-bodied animals, and it is unknown how body size evolution has impacted feather distribution and development, although a recent model-based analysis suggests the small theropod *Coelophysis* might have required filamentous integumentary structures for insulation [66].

Simple branched morphologies have been reported in a few pterosaurs and ornithischians and in many early diverging tetanuran theropods, but in some cases more evidence is required to confirm that their branched morphologies are genuine, rather than resulting from preservational artefacts, such as confusion with overlapping monofilaments or the decomposition of fibre-like structures [67]. In compressed fossil feathers, such as those accompanying many of the most spectacular non-avialan avemetatarsalian and avialan fossils, complete morphologies are sometimes difficult to observe [20,65]. Feathers in amber provide higher fidelity morphology than compressed fossil feathers [17,68,69], but they are often disassociated from the host animals. So far, there is only one confirmed amber specimen preserving non-avialan theropod feathers, but even in this case, the phylogenetic position of this fossil is uncertain [17], limiting its contribution to broader evolutionary discussions.

Some developmental features have been observed in early diverging pennaraptoran theropods, including the delayed development of secondary remiges, unequal development of remiges and rectrices, and the presence of strikingly different morphotypes during the development of remiges and rectrices in one oviraptorosaurian taxon [49], although this unusual morphotype was identified as pin feathers in some studies [65,70,71]. Early birds also display some plumage developmental features unusual for modern feathers [72]. One hatchling enantiornithine bird specimen has been suggested to be unusual in possessing relatively poor body feathering but well-developed remiges, with its first generation of feathers being more contour-feather-like than down-like [73], although similar features are also seen in some living birds [74]. Several enantiornithine fossils show pin feathers widely distributed on the body, including the trunk [71]; and some enantiornithines developed ornamental feathers early in ontogeny [75], probably as early as the first postnatal moult [73]. Collectively, these fossils demonstrate that there are developmental differences between early feathers and modern feathers, and the processes controlling early feather formation seem to be less constrained and more flexible than for modern taxa [49].

At the ultrastructural and molecular levels, fossil feathers show some potentially interesting features. For example, the filamentous feathers of the alvarezsaurian *Shuvuuia* lack feather-type corneous beta proteins and these proteins form only a small proportion of the pennaceous feathers in *Anchiornis*, both in stark contrast to modern feathers where feather-type beta proteins (i.e. β-keratins) dominate [76]. However, it has been questioned whether immunohistochemical methods are applicable to these fossils [77], and the recovered molecular features might not be original.

Solving these conflicts and uncertainties will require: in-depth analyses of the filamentous integumentary appendages of ornithischians and pterosaurs; the development of new geochemical and immunohistological assays that are applicable to fossil material; and further work to identify follicles or their precursors in feathered and non-feathered taxa. Most importantly, however, we need more fossils with soft-tissue preservation to determine whether integumentary appendages were present or absent in various key clades.

## 4. Future directions

The key outstanding questions in feather evolution remain: (i) the timing of feather origin; and (ii) the homologies of pterosaur, theropod and ornithischian integumentary appendages. The two competing scenarios for feather origin, i.e. the ‘avemetatarsalian’ and ‘tetanuran theropod’ hypotheses, imply significantly different phylogenetic and temporal frameworks for feather evolution. The former is based on the assumption that pterosaur and dinosaur feathers are homologous, which optimizes their origin close to the base of Avemetatarsalia in the earliest part of the Triassic, around 245 Ma [52,57]. The latter hypothesis regards pterosaur and ornithischian integumentary structures as potentially non-homologous and restricts ‘true’ feather evolution to a subgroup of the theropod dinosaurs, suggesting an Early Jurassic origination of feathers as well as independent acquisition of feather-like structures [55,58].

Most significantly, these two hypotheses ask different ‘why’ questions. If correct, the avemetatarsalian hypothesis needs to account for why feathers are so frequently lost in avematatarsalian evolution (in many ornithischians and sauropodomorphs and ‘naked’ pterosaurs lacking clear integumentary coverings). Are these losses related to different developmental mechanisms for early feathers and modern feathers? Or is their loss related to the fact that some non-avialan dinosaurs had differing physiologies from modern birds? Were either the evolution of large body size or the invasion of different thermal niches potential drivers? Also, can the unusual structures of some ornithischian appendages be reconciled with known theropod/bird developmental pathways? Answering these questions will require explanations from both developmental and evolutionary perspectives.

Conversely, the tetanuran origin hypothesis needs to explain why multiple avemetatarsalian lineages evolved filamentous integumentary appendages at different times, and whether there are fundamental structural or developmental differences between ‘true’ feathers and the filamentous integumentary appendages of pterosaurs and ornithischians. While it might be true that feathers can be only optimized as a synapomorphy near the base of the tetanuran theropods under some evolutionary models, in terms of deep homology it is possible that a common genetic regulatory network might have originated at the base of the Avemetatarsalia given the similarities in the filamentous integumentary appendages in pterosaurs and ornithischians [55,58].

Neontological studies also provide some support for this deep homology scenario. Modern feathers are biochemically similar to some other archosaurian integumentary structures [78–80] given that feather-type corneous beta proteins are also present in avian scutate scales, beaks and claws, the non-follicular bristles of the wild turkey ‘beard’, as well as alligator claws and embryonic scales, though feather-type corneous beta proteins are slightly larger in size in scales than in feathers [23,78–80]. Neontological studies have also revealed surprising cellular-level similarities between avian feathers and embryonic scutate scales and the embryonic epidermis of *Alligator* [45,79–82]. These studies provide support for the similar developmental mechanisms for the integumentary system, particularly the filamentous integumentary appendages of dinosaurs and pterosaurs. However, they also show how these mechanisms can generate scale-like morphologies. Thus, it is important to investigate what molecular and developmental mechanisms are responsible for the key features differentiating feathers from scales.

One other key research direction is the development and evolution of integumentary appendage distribution pattern. On the one hand, avian integument is an integrative system: modern feathers are associated with avian skin, which dramatically differs from typical reptilian skin [1,2]; conversely, avian integument is heterogeneous and displays regional specification: most parts of the body are covered by various feathers, other parts by different scale types, and some parts are naked [1]. Feathers also display distinct distribution patterns to form feather tracts [1]. When did distinct integumentary heterogeneity and/or feather tracts appear? Do the integumentary heterogeneity patterning and feather tract patterning remain unchanged or have they evolved since they first appeared? Are feather development and avian skin anatomy always coupled developmentally and evolutionarily?

Recent studies suggest that at least some feathered non-avialan maniraptorans and enantiornithine birds had skin that is structurally similar to that of extant birds [83,84], and it is important to investigate the skin of other non-maniraptoran avemetatarsalians to determine if it is morphologically closer to that of living birds or reptiles. One recent study on a new specimen of *Psittacosaurus* showed that scaly patches of skin had reptilian structure [85], and it is imperative to investigate the skin features of the ‘quilled’ region of the earlier-described Senckenberg specimen [61]. Evidence of avian-type skin structure in the body regions of an ornithischian and/or pterosaur-bearing filamentous appendages would provide additional support for the avemetatarsalian origin hypothesis, whereas reptilian skin features in these regions would undermine it. Although some (but not all) pterosaurs display filamentous integumentary appendages [57,63,64], all pterosaurs with soft-tissue preservation seem to lack scales, suggesting that their bodies were mostly naked and leathery, indicating more possible instances of feather loss [58]. The ornithischians *Kulindadromeus* and *Tianyulong* possess extensive integumentary appendages over some body regions but scaled tails [56,59,60], whereas *Psittacosaurus* displays an extensively scaled body, partially scaled tail and quill-like tail appendages [61,62,86], but most other ornithischians are extensively scaled [58,86,87]. Sauropodomorphs probably had scaled skin [58,88]; whereas many tetanuran theropods (except some large taxa such as tyrannosaurids) are extensively feathered; single filaments are restricted to certain regions in some theropods such as *Beipiaosaurus* [29]; and some coelurosaurian theropods have extensively feathered lower legs and even feet [89], in contrast to the scaled foot in most modern birds [1]. While it is possible that all avemetatarsalians were partially feathered and partially scaled, many non-avian taxa show significant differences in scale and feather patterning on the body from that seen in extant birds. These observations give rise to several additional questions that require resolution. When did feathers become the dominant integumentary appendages? Is there is flexible evolutionary stage that is characterized by significantly different degrees of body feathering in different avemetatarsalian taxa? When did secondary coverings of scutate and reticulate scales—the two main types of pedal scales in birds: the former resembling the overlapping scales of reptiles and possessing feather-type corneous beta proteins and the latter are similar to the dome-shaped reptilian tuberculate scales— appear? When were primitive ‘reptilian’ scales finally lost? Were pterosaurs primitively naked?

Finally, some of current debate derives from different ways to define a feather. Modern feathers are defined by a set of unique biochemical, developmental and morphological features [4,22–26], which have been used to identify early feathers (e.g. [4,23]). Because these features have probably evolved sequentially among early feathers, some of them are likely to have been absent in the earliest feathers. For example, it is possible that the earliest feathers were filaments without an associated follicle (e.g. [40]), even though a follicle is a key criterion for the identification of modern feathers (see above). Identifying modern and early feathers is somewhat comparable to identifying crown group and stem group birds, and thus the identification of the earliest feathers could become an issue of researchers’ choice even though phylogenetic continuity could establish homological relationships between fossil integumentary structures and modern feathers. More broadly, it is important to study the development and evolution of feathers and closely related integumentary structures (e.g. turkey beard and avian scutate scales) and set this information in a phylogenetic context, paralleling the situation in research on bird origins, where the emphasis has shifted from focusing on bird cladogensis to considering broader evolutionary events and changes in key morphological systems (e.g. [53]).

## 5. Conclusions

Integumentary data from neontological morphology, developmental biology and comparative genomics are important for understanding feather morphogenesis, development and functions [22,90–93], but fossil data are playing a key role in reconstructing the evolutionary history of feathers. However, new fossil data are required from in-depth analyses of old specimens or new specimens in poorly sampled areas of the avemetatarsalian phylogenetic tree to provide the evidence needed to test different models of feather evolution. Given the known diversity of skin appendages in extinct avemetatarsalians, and the possibility that others remain to be found, these palaeontological discoveries also shed light on novel aspects of feather evolution unseen in modern avian taxa and have the potential to open new and surprising lines of enquiry.

## Data Availability

This article has no additional data.
